# A brief history of crystalloids: the origin of the controversy

**DOI:** 10.3389/fped.2023.1202805

**Published:** 2023-07-03

**Authors:** Jaime Fernández-Sarmiento, Carolina Casas-Certain, Sarah Ferro-Jackaman, Fabian H. Solano-Vargas, Jesús Ángel Domínguez-Rojas, Francisco Javier Pilar-Orive

**Affiliations:** ^1^ Department of Critical Care Medicine and Pediatrics, Universidad de La Sabana, Fundación Cardioinfantil-Instituto de Cardiología, Bogotá, Colombia; ^2^ Department of Pediatrics, Universidad del Rosario, Fundación Cardioinfantil-Instituto de Cardiología, Bogotá, Colombia; ^3^ Department of Critical Care and Emergency, Instituto Nacional de Salud del Niño, Lima, Perú; ^4^ Department of Pediatrics and Critical Care, Biocruces Bizkaia Health Research Institute, Cruces University Hospital, Bilbao, Spain

**Keywords:** fluids bolus, sepsis, children, resuscitation, shock

## Abstract

Fluid resuscitation with crystalloids has been used in humans for more than 100 years. In patients with trauma, sepsis or shock of any etiology, they can help modify the clinical course of the illness. However, these solutions are medications which are not side-effect free. Recently, they have been questioned in terms of quantity (fluid overload) and their composition. The most frequently used crystalloids, both in high and low-income countries, are 0.9% normal saline (NS) and Ringer's lactate. The first descriptions of the use of sodium and water solutions in humans date from the cholera epidemic which spread throughout Europe in 1831. The composition of the fluids used by medical pioneers at that time differs greatly from the 0.9% NS used routinely today. The term “*physiological solution”* referred to fluids which did not cause red blood cell hemolysis in amphibians in *in vitro* studies years later. 0.9% NS has an acid pH, a more than 40% higher chloride concentration than plasma and a strong ion difference of zero, leading many researchers to consider it an unbalanced solution. In many observational studies and clinical trials, this 0.9% NS composition has been associated with multiple microcirculation and immune response complications, acute kidney injury, and worse clinical outcomes. Ringer’s lactate has less sodium than plasma, as well as other electrolytes which can cause problems in patients with traumatic brain injury. This review provides a brief summary of the most important historical aspects of the origin of the most frequently used intravenous crystalloids today.

## Introduction

Crystalloids are medications which contain fluids and electrolytes to help maintain the body's fluid balance (1–3). They have been widely used in medicine for more than 100 years due to their low cost, wide availability and effectiveness in intravenous hydration and fluid resuscitation (4, 5). Historically, the early and “*aggressive*” administration of crystalloids in adults and children has been supported for patients in shock. This practice has led to early recognition of critically ill patients and changes in mortality in high and low-income countries (6–8). However, it has been recently recognized that crystalloids are not innocuous. They have been questioned in terms of excess volume (quantitative) and their composition (qualitative) (9–11). Excessive fluid administration and very positive balances have been associated with worse outcomes in critically ill patients (12–14). In addition, the composition of some crystalloids, like 0.9% saline solution, has been questioned due to their acid pH (5.0) and excessive chloride (40% more than plasma). Studies from more than 100 years ago as well as some recent ones have warned of the complications associated with their use (9, 15–18). The association between the use of 0.9% NS and acute kidney injury and microcirculation changes has alerted researchers and clinicians to consider alternatives for crystalloids in patients with circulatory abnormalities (9, 11, 19–22).

Thus, the ideal choice of crystalloids for fluid resuscitation is a matter of controversy today. In adults, several clinical trials have been conducted with a significant number of patients. A recent systematic review and meta-analysis found that there may be a small relative reduction in mortality when balanced solutions are used compared with 0.9% NS (15). Balanced solutions have been found to produce a relative reduction of 9%, or a 1% increase, in 90-day mortality. This figure suggests that, overall, it would seem reasonable to consider that using balanced solutions is associated with a reduction in mortality. Although this impact on mortality could be considered “relatively” small, we must remember that crystalloids may be one of the most used medications in medical practice. In this context, a small reduction could be significant when it is used so frequently as a resuscitation fluid (15, 23, 24).

Remembering the most important aspects of the origin of intravenous crystalloids can help us better understand the current questions. The process of developing, producing and storing these solutions has changed significantly over time (2). Likewise, their indications, volume and administration are increasingly tailored to the patients' needs (10). One more step toward this interesting path of precision medicine which, on this particular topic, can significantly impact the outcomes of critically ill patients. It may be that the use of both 0.9% NS and Ringeŕs lactate should be individualized. Taking specific patient aspects into account (comorbidities, electrolyte levels, and hemodynamic coherence, among others) could help in selecting a crystalloid with fewer adverse effects. In addition, it should be noted that crystalloid solutions with low dextrose concentration are available in some countries. This could be important, especially in infants with low liver glycogen reserves who may have simultaneous hypoglycemia or other associated disorders like hyponatremia. In this brief review, we seek to recall the process which has led people to use crystalloids in clinical practice, particularly for fluid resuscitation, and thus understand a bit better the origin of some of the controversies and complications associated with their use.

### Unbalanced solutions: A case called 0.9% saline solution

The cholera epidemic in 1830 was one of the most devastating pandemics of modern history. Originating in India in 1817, the disease spread rapidly around the world, reaching Europe in 1830 (17). Cholera spread throughout France, Italy, Spain and the United Kingdom, killing more than 100,000 people. The disease was especially aggressive in London, where a lack of sanitation infrastructure and unsanitary living conditions contributed to the rapid dissemination of the disease. In 1832, the British physician Thomas Latta proposed that cholera could be treated with a water and salt solution administered by a route other than the oral or rectal route (18, 25).

**Figure 1:**
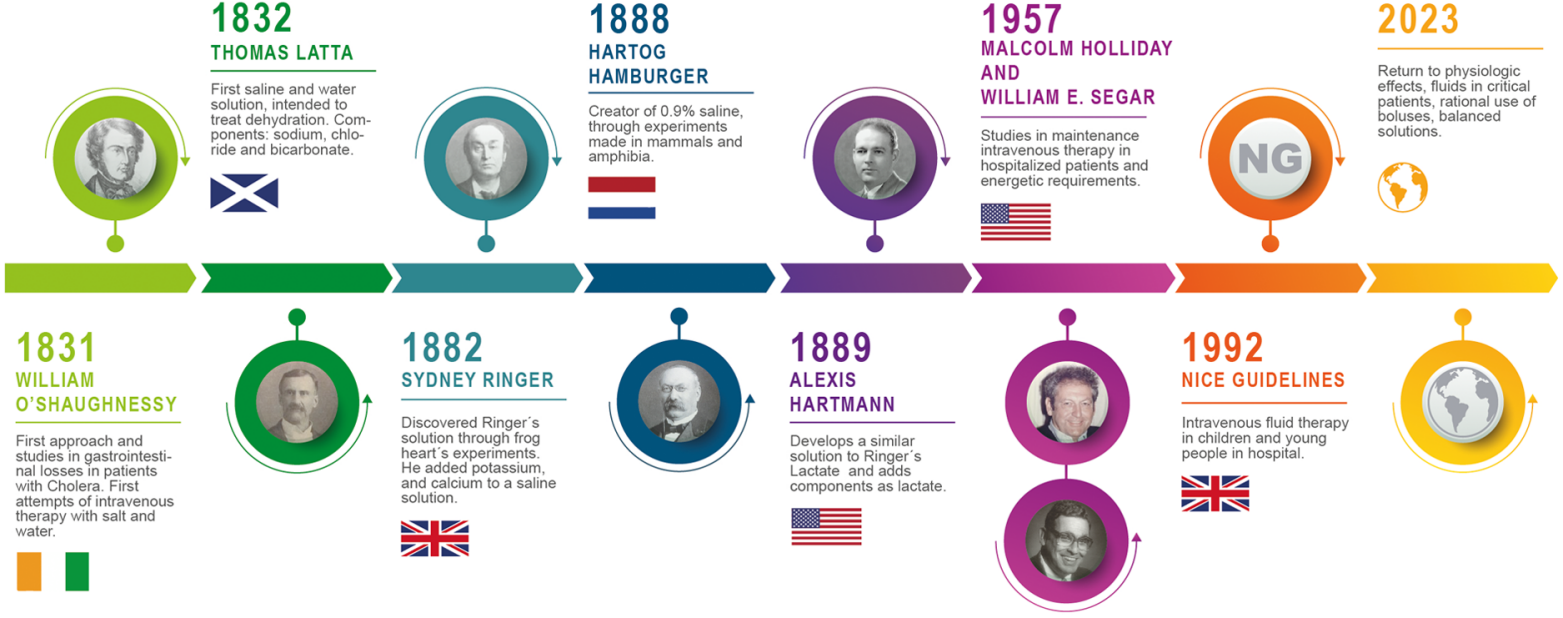
Timeline of the evolution of crystalloid solutions.

Latta's solution was made up of sodium chloride, sodium bicarbonate and distilled water (Figure 1 gives a summary of the historical development of crystalloids and the main contributions of the researchers). It was administered intravenously, which was a novel strategy at that time. Although Latta's solution was effective for hydrating patients, it was also toxic at high doses, due to its fluid and electrolyte makeup (26). Latta conducted his first experiment on a middle-aged woman who had received all the treatments considered to be effective for cholera at that time. There was no response, and the woman died after receiving this solution intravenously (25). However, Latta continued his studies and conducted experiments in animals with different concentrations of sodium and chloride, originating the first mixture of water and sodium for intravenous use.

With the end of the cholera epidemic, saline solution could have been consigned to the history books. In 1896, the Dutch chemist Hartog Jacob Hamburger created a solution he termed “*physiological serum*” to study the hemolysis of red blood cells *in vitro*. He never intended to use this solution clinically (1, 4, 27). His experiments consisted of mixing solutions with different tonicities and evaluating the interaction of these solutions with erythrocytes from various species with regard to changes in concentration and temperature. Using the freezing point of amphibian and mammalian blood, Hamburger concluded that “*warm blood*” and 0.9% NaCl solution had similar freezing points. In addition, when erythrocytes had contact with this solution, it did not induce hemolysis, and therefore could be considered isotonic with warm-blooded mammals. Hamburger considered this solution to be “*normal*” or “*physiological*” since it did not cause hemolysis, as did hypotonic solutions. Experiments in amphibians showed that a solution with a 0.6% sodium concentration could be considered “indifferent” or “physiological” for frogs, because it did not cause hemolysis. Since that time, solutions with a sodium and water content which did not cause red blood cell lysis in amphibians or mammals were called “*physiological solutions*” (28, 29).

Around this same time, towards the end of the 19th century, the researcher Frances Alexis Carrel had begun to work on what could be the conservation of human tissue (30). As he was interested in vascular suturing and organ transplantation, he began to work on a solution that would sustain human tissue outside of the body. Based on Latta's experiments, he designed a sodium chloride solution with a content similar to plasma. With this solution, he was able to sustain human tissue to continue his transplant experiments. Years later, he received the Nobel Prize in Physiology. The editorial in Lancet on October 19, 1912, stated: “*And there is a new advance in blood vessel surgery which is, perhaps, even more surprising. Carrel has demonstrated that a portion of the artery can be kept in cold storage for several days or even weeks before the transplant and, even so, stay alive. No one who has followed these new surgical advances with interest can doubt that they have immense potential, and that the application of the methods learned in animals to human beings cannot take long…”*

The idea of using 0.9% NS in clinical practice resulted from experiments in dogs with intestinal obstruction. Hartwell and Hoguet applied subcutaneous of “*normal saline solution*” in their study of animals with intestinal obstruction (31). In 1913, Truch et al., based on these descriptions, proposed that fluids could be replaced after surgery in some patients through “*proctoclysis*.” They compared almost 2,000 adults who received tap water vs. “normal” saline solution enemas. They warned of the risks of administering a solution with a high sodium and chloride content: “*we would be forcing an already weakened patient, in the space of 24 h, to receive the average amount of salt eaten as a condiment by a normal person in a month.”*

**Figure 2:**
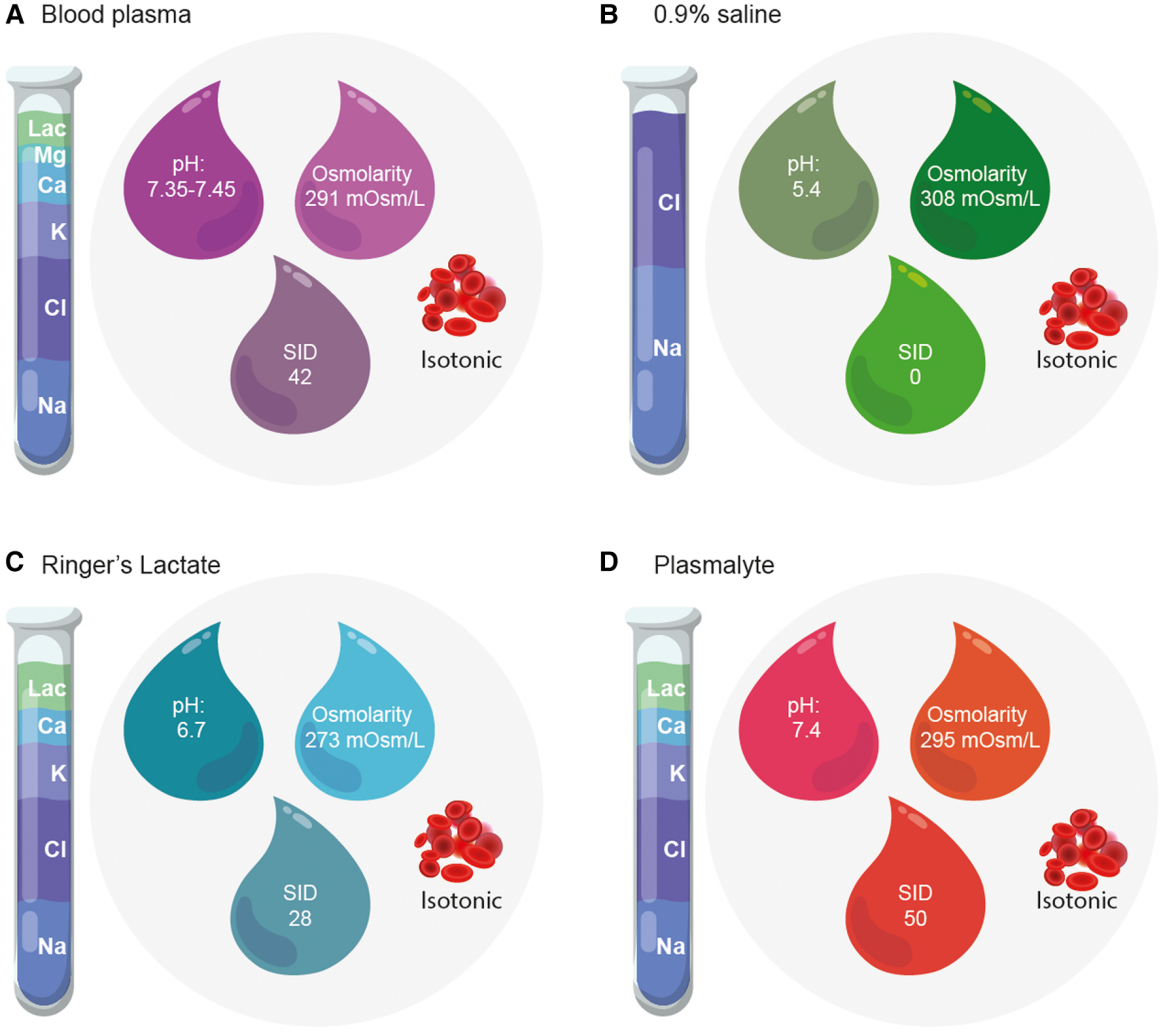
Composition of blood plasma and crystalloid solutions. The composition of crystalloids and blood plasma differ in terms of electrolyte concentration, pH, osmolarity and strong ion difference. (**A**) Composition of Blood plasma. (**B**) Composition of 0.9% saline. (**C**) Composition of Ringer's Lactate. (**D**) Composition of Plasmalyte-148 ©.

Despite these descriptions of almost 100 years ago, 0.9% saline solution continues to be called “*physiological*,” even though its composition does not resemble the composition of plasma (Figure 2). 0.9% saline solution has approximately 10% more sodium than plasma, 40% more chloride, an acid pH, a strong ion difference (SID) of zero. The SID is the total difference between strong anions and cations. It is simplified as the difference between sodium and chloride. Normal plasma is considered to have an SID between 38 and 42 mEq/L. This difference is associated with acidosis when large volumes of chloride are administered such as, for example, with the use of large amounts of 0.9% saline (18, 20).

Only Hamburger's experiments indicate that this crystalloid does not cause red blood cell hemolysis. It remains a mystery how it came to be a general-purpose *in-vivo* intravenous liquid. Perhaps it was due to the ease, convenience and low cost of mixing common salt with water. With the current evidence, the use of unbalanced solutions is once again being questioned. Animal and human models have found that the composition of 0.9% NS has been associated with a greater inflammatory response, immunological disorders, endothelial activation and glycocalyx degradation, among other problems. All of which have a clinical impact in the greater frequency of hyperchloremic metabolic acidosis, kidney failure, and mortality associated with its use (9, 22, 32–34).

### Balanced solutions: Ringer's lactate and multiple electrolytes solution

Sidney Ringer was a 19th century British physiologist known for his studies on metabolism and cellular function. One of Ringer’s most famous experiments was his study on the chemical composition of extracellular fluid and its influence on muscle contraction (35, 36). His studies were mainly done in amphibians. His experiments consisted of instilling distilled water with inorganic salts into frog hearts and evaluating aspects like contractility. After his assistant made a mistake in one of the experiments, tap water was used instead of distilled water. Ringer was surprised to see the cardiac behavior in frogs and their ability to survive. Thus, he described the importance of extracellular water and electrolytes in the cardiac work of living beings. This experiment laid the foundation for the modern understanding of homeostasis and cell regulation. In his observations, he emphasized the need for appropriate proportions of potassium, calcium and chloride for protoplasmic activity. Therefore, he proposed a mixture of water and electrolytes which he called “*Ringer’s solution.*” He considered that this solution and its electrolyte proportions could temporarily “*replace”* blood and provide a physiological medium for its adequate functioning (36).

Ringer’s solution contained a liter of distilled water, sodium chloride, 0.33 g (mEq) and calcium. This solution had a higher pH than 0.9% NS and less chloride (2, 35). Subsequently, around 1932, the American pediatrician and biochemist Alexis Hartmann modified Ringer's solution (37, 38). He added sodium lactate in order to reduce the acidosis found in infants with diarrhea, dehydration and oliguria. Since then, Ringer's lactate has been considered to have a composition more like that of human plasma. However, although its use in large quantities has been questioned due to its potassium and lactate content, it has not been shown to have serious side effects that would require its suspension in patients with kidney injury or liver dysfunction (39–41). In fact, in a clinical trial in adults following kidney transplantation, Ringer's lactate was associated with less acidosis and hyperkalemia (42). In addition, it has been used as a solution for diluting human albumin (reducing a 20% concentration to 4%), proving to be safe and isotonic, possibly related to negative charges in the gelatin molecules contributing to plasma osmolality (43). However, this solution should not be used in children with head trauma because it has been associated with worse outcomes and even increased mortality (44).

Other balanced solutions have been developed recently. The use of intravenous fluids for maintenance therapy or fluid resuscitation in children in critical care is a universal practice. Multiple electrolytes solutions that are more similar to plasma in all its components are increasingly wanted. This is the case of Plasma-Lyte 148® (PL-148). This is a crystalloid which was patented in 1982 by Baxter International Inc® (45). They were seeking a solution which would have the physicochemical properties of plasma and could truly be considered “physiological.” It contains 140 mmol/L of sodium, 5 mmol/L of potassium, 1.5 mmol/L of magnesium, 98 mmol/L of chloride, 27 mmol/L of acetate and 23 mmol/L of gluconate (46). Its composition gives it a pH of 7.4, which is adjusted with sodium hydroxide. The term “148” comes from the sum of its cations (sodium, potassium and magnesium). PL-148 is safe for diluting medications commonly used in intensive care (morphine, fentanyl, ketamine, salbutamol, aminophylline, and clonidine are stable for 24 h when mixed with Plasma-Lyte 148® and Plasma-Lyte 148® + 5% Glucose for administration at equivalent concentrations) (45). Clinical trials in children have shown that PL-148 is safe and effective when compared with other crystalloids (46). Its greatest benefits may be related to its physiological chloride content and better pH than other solutions, with a similar cost to other crystalloids in most countries where it is available (47).

### Understanding the controversy better: physicochemical aspects

Osmolality is the concentration of particles dissolved in a liquid. In medical science, osmolality is used to determine certain serious conditions like diabetes, dehydration and shock (48). Plasma osmolality ranges from 280 to 296 mOsmol/kg. The concentration of substances like chloride, sodium, potassium, glucose and urea are calculated. The solvent volume remains the same regardless of changes in pressure or temperature. The common method for measuring osmolality is through osmometry. The osmotic activity of crystalloids and intravenous fluids is best described by calculating the *in vivo* osmolality (mOsm/kg) of the solution (48, 49).

It is important not to confuse osmolality with osmolarity. Osmolarity is the concentration of a solute. It corresponds to the number of osmoles of solute particles per unit volume of solution (10). The osmotic pressure of a solution determines the solvent's diffusion through a semi-permeable membrane separating solutions with different osmotic concentrations. Neither should this concept be confused with tonicity (49). Tonicity is part of the solution's total osmolarity. It is the force exerted by the particles which do not freely pass through the membrane. Therefore, tonicity can be described as the “*relative concentration*” of the solution. Thus, the term “osmolarity” is the total concentration of diffusible and non-diffusible solutes. Tonicity is the total amount of only non-diffusible solutes.

*In vitro* 0.9% NS is slightly hypertonic, with an osmolality of 308 mOsmol/kg (154 mOsml/kg of Na^+^, 154 mOsml/kg of Cl^−^) (48). However, as these electrolytes are only partially active, *in vivo* 0.9% NS has a calculated osmolality of 287 mOsmol/kg. That is, it is considered isotonic due to its osmolality. Often, the term “isotonic” is confused with “physiological.” The first refers to its osmolality, the second to its composition. 0.9% NS is isotonic, but its composition is not physiological. *In vivo* Ringer's lactate has an osmolality of approximately 274 mOsmol/kg (slightly hypotonic) and PL-148 has an osmolality of 270–290 mOsmol/kg. To avoid confusion between tonicity and composition, it is preferable to classify crystalloids as balanced or unbalanced (10, 48, 49).

The Pragmatic Pediatric Trial of Balanced vs. Normal Saline Fluid in Sepsis (PRoMPT BOLUS) is currently being conducted (50). This clinical trial compares fluid resuscitation using balanced vs. unbalanced solutions in children with sepsis. With a large sample size (slightly more than 5,000 patients), this study will provide high-quality evidence of the possible differential effects of these crystalloids. For now, with the available observational data and trials in children, the clinical trials in adults and the weak historical support for the use of 0.9% NS, it would seem reasonable to consider balanced solutions to generally be the best available option for fluid resuscitation in patients who require it (51). However, each case should be evaluated individually. All the factors which may affect patient outcomes should be considered when ordering crystalloids, to ensure they are a safe approach. This is one more step toward precision medicine in fluid resuscitation. For example, patients with head trauma or hyponatremia should receive 0.9% NS as the crystalloid of choice for fluid resuscitation, because balanced solutions have been associated with worse outcomes. For children with septic shock, the adult and pediatric sepsis consensuses recommend the use of balanced solutions as the liquids of choice (6, 8).

## Conclusions

The indiscriminate use of crystalloid boluses is being questioned in terms of their quantity and quality of composition. Intravenous fluids were established in clinical practice and licensed for use without robust investigation of their efficacy or safety.

0.9% NS has a historical basis which suggests that its development was aimed at *in vitro* experiments rather than use in humans. The term “*physiological solution*” should be considered inappropriate for this solution due to its composition. Balanced solutions have a composition more similar to plasma, and recent evidence suggests that they may have fewer side effects. The decision to perform fluid resuscitation should include an appropriate choice of the type of crystalloid to be used, with an individualized approach, considering the potential risks and complications associated with their use. The precision medicine based strategy for fluid resuscitation should be the fundamental principle of treatment for all patients, considering the best crystalloid (balanced or unbalanced) according to each particular case.
